# Improvement of anxious but not depressive symptoms is associated with functional outcomes of psychocardiological rehabilitation

**DOI:** 10.1038/s41598-025-32594-7

**Published:** 2025-12-26

**Authors:** Lilia Papst, Christoph Schmitz, Eike Langheim, Volker Köllner

**Affiliations:** 1 Psychosomatic Rehabilitation Research Group, Department of Psychosomatic Medicine, Center for Internal Medicine and Dermatology, Charité University Medicine Berlin, Berlin, Germany; 2 Department of Cardiology, Rehabilitation Centre Seehof, Teltow, Germany; 3 Department of Psychosomatics and Behavioral Psychotherapy, Rehabilitation Centre Seehof, Teltow, Germany

**Keywords:** Psychocardiological rehabilitation, Depressive disorders, Anxiety disorders, Cardiovascular disease, Heart-focused anxiety, Cardiology, Cardiovascular diseases, Depression, Anxiety, Quality of life, Rehabilitation, Outcomes research

## Abstract

Cardiovascular and mental health conditions are closely linked, and integrative treatments increasingly target both. However, it remains unclear which psychological improvements most strongly influence functional outcomes, such as work ability and quality of life. We hypothesized that reductions in depressive symptoms, general anxiety, and heart-related anxiety would be associated with better work ability, fewer work-related impairments, and improved quality of life. We assessed 243 patients undergoing psychocardiological rehabilitation at admission and discharge. Depressive and anxiety symptoms were measured using the Hospital Anxiety and Depression Scale (HADS), heart-related anxiety by Cardiac Anxiety Questionnaire (CAQ), work-related impairments by sociomedical evaluation and the Mini-ICF-APP Social Functioning Scale (MINI-ICF-APP-S), and quality of life with the Short Form-12 health survey (SF-12). Regression analyses revealed that reductions in general anxiety (HADS-A) and heart-related anxiety (CAQ) were linked to fewer work-related impairments (*p* = 0.03) and higher mental quality of life (*p* ≤ 0.001 and *p* ≤ 0.01, respectively). Reduced heart-related anxiety was also associated with higher physical quality of life (*p* ≤ 0.001). In contrast, higher baseline depressive symptoms (HADS-D) increased the odds of limited work ability (OR = 1.78, *p* = 0.01), while improvements in depression were not associated with functional outcomes. These results suggest that depressive and anxiety symptoms are similarly responsive to psychocardiological rehabilitation, but only the latter are associated with improved functional outcomes.

**Supplementary Information:**

The online version contains supplementary material available at 10.1038/s41598-025-32594-7.

## Introduction

Cardiovascular and mental health conditions frequently show comorbidities^1,2^. In particular, major depression and anxiety disorders occur with high global lifetime and high point prevalences in populations with cardiovascular disease (CVD)^3^.

Indeed, major depression was associated with a pooled 72% increase in risk for developing a cardiovascular disease and a point prevalence of 18% in patients with CVD^3^. Depression and cardiovascular diseases have a range of shared behavioral and psychosocial risk factors such as physical inactivity, smoking, catastrophizing thoughts, low stress resilience, low socioeconomic status, and early-life stress, alongside shared biological risk factors involving inflammation, autonomic dysregulation, and genetic factors^4–7^. Moreover, depression not only increases the risk for cardiovascular diseases such as coronary artery disease, heart failure, and arrhythmias^8,9^, but also influences disease progression, treatment adherence, and rehabilitation outcomes. That is, it reduces uptake and retention in rehabilitation programs, undermines adherence to prescribed behavioral and exercise-based therapeutic regimens, and attenuates improvements in physiological markers (e.g. cardiopulmonary fitness, body composition) that are themselves predictive of longer-term cardiovascular outcomes.^10^.

Anxiety disorders on the other hand increase the risk of developing a cardiovascular disease by 41% and affect approximately 11–29% of cardiology patients^3,11^. Similarly to depressive disorders, the relationship is considered bi-directional, i.e. anxiety may increase the risk of developing cardiovascular conditions^12^ and vice versa^13^. Indeed, anxiety following major cardiac events can lead to heightened bodily vigilance, fear of physical activity, and avoidance behaviors^14–17^, further complicating recovery.

From the economic perspective, it is worth noting that heart disease and psychiatric disorders are among the leading causes of disability worldwide^18,19^. Thus, it is imperative to provide these patients with tailored rehabilitation programs that facilitate their return to work and societal reintegration. However, the treatment of relevant psychiatric comorbidities in patients with cardiovascular disease has rarely been addressed in existing rehabilitation concepts making for an often-unsatisfactory decision between two monodisciplinary concepts for referring physicians.

Psychocardiological rehabilitation was specifically designed for patients with comorbid psychosomatic disorders (e.g., depression, anxiety, PTSD) and cardiological diseases (e.g., coronary heart disease, arrhythmia, structural heart defects), both of which require rehabilitative specialist diagnosis and treatment^20^. This includes patients with specific cardiac arrhythmias and a resulting anxiety disorder with avoidance behavior, patients with coronary heart disease and comorbid depressive disorder, patients with PTSD as a result of severe cardiovascular disease, or patients with severe adjustment and adherence disorders related to cardiovascular disease. Table 1 gives an overview of the types of rehabilitation and examples for indication^21^.

**Table 1:** Indications for rehabilitation.

Type of rehabilitation	Patient example
Cardiological rehabilitation	Minor depressive symptoms and insecurities after cardiac operation or infarct. No need for patient education, intensive psychological support or psychotherapy
Psychosomatic rehabilitation	Mental health problems are central with no existing heart disease indicating a need for rehabilitation, i.e. panic attacks and heart anxiety in patients with a healthy heart, depressive disorder with a history of a heart attack in the past
Psychocardiological rehabilitation	PTSD after an implantable cardioverter-defibrillator shock series, manifest depressive or anxiety disorder after myocard infarct or heart insufficiency, complicated arterial hypertonia with strong adherence problems, problematic coping with illness due to congenital heart defects or after heart transplantation

To date, rehabilitation clinics using a “dual” psychocardiological rehabilitation approach have predominantly used concepts developed in-house. An initial concept for psychocardiological rehabilitation now allows for the development of therapy standards for cost bearers and rehabilitation clinics^20,22,23^. It combines elements of both cardiac^24^ and psychosomatic rehabilitation^25^, with a focus on interdisciplinary diagnostics, exercise therapy, and concurrent cognitive-behavioral psychotherapy. There are currently ongoing EU-wide evaluation efforts of psychocardiological rehabilitation^26^. We here present our most recent findings from a secondary analysis of the “Effectivity of Psycho-Cardiological Rehabilitation (EvaPK)” study which is registered in the International Clinical Trials Registry Platform under the ID DRKS00023370.

We would like to add to the growing research body by looking at which psychological symptom changes are associated with functional outcomes at the end of psychocardiological rehabilitation, which to our knowledge has not yet been reported. While the most obvious goal for any health intervention is symptom improvement, health extends far beyond the mere absence of complaints, and rehabilitation efforts are generally aimed at the much broader goal of societal participation. In western civilizations this is usually associated with pursuing a gainful employment.

This is why our primary outcome is the subjective and objective capacity to pursue work after completing psychocardiological rehabilitation. In addition, we were interested in how symptom improvements affected overall quality of life, which itself is inversely related to early retirement^27,28^. Improvements in heart anxiety, general anxiety and depressive symptoms were defined as explanatory variables due to depression and anxiety being the most frequent comorbid psychiatric diagnoses for cardiac diseases^3^.

Specifically, we hypothesized that reductions in symptom load in general anxiety and heart anxiety, as well as depression significantly and independently related to lower work-related impairments, a lower chance for reduced work ability, as well as improved quality of life at discharge from psychocardiological rehabilitation.

## Methods

Data were obtained between April 2019 and March 2021 as part of the research project “Effectivity of Psycho-Cardiological Rehabilitation (EvaPK),” which is registered in the International Clinical Trials Registry Platform under the ID DRKS00023370. Approval was granted by the ethical committee of the State Medical Board of Brandenburg, Germany, on Jan. 8th, 2019 (No. S1(a)/2019) and the study was conducted in compliance with the Declaration of Helsinki^29^.

### Study design

The current investigation was a secondary analysis of data collected for the EvaPK study. For this purpose, we analyzed a subsample of patients with depressive and anxiety disorders in the psychocardiological rehabilitation group at admission and discharge in an observational longitudinal pre-post design.

### Participants

Participants were recruited in a rehabilitation clinic of the German Pension Fund in Teltow, Germany, and asked for written informed consent. Inclusion criteria were an age between 18 and 70 years and the presence of a somatic cardiac diagnosis as well as a psychiatric diagnosis of the categories affective disorders, neurotic, stress-related and somatoform disorders or psychological and behavioral factors associated with disorders or diseases classified elsewhere. Exclusion criteria were acute psychotic or manic disorders, severe cognitive impairments or the inability to comprehend diagnostic instructions or questionnaires. We analyzed the data of N = 243 inpatients in psychocardiological rehabilitation with a diagnosis of depression (n = 91), anxiety disorder (n = 49), or both (n = 103).

### Diagnoses

Both psychological and cardiological diagnoses were initially made by referring physicians according to ICD-10 criteria as a prerequisite for the patient to be granted access to psychocardiological rehabilitation. The clinical diagnoses were independently verified in-house by the attending physician who rated the patients according to AMDP (“Arbeitsgemeinschaft für Methodik und Dokumentation in der Psychiatrie”, a German-language Consortium for Methodology and Documentation in Psychiatry) and ICD-10 criteria, validated by a second senior physician in accordance with the dual control principle.

### Psychocardiological treatment program

Psychocardiological rehabilitation has a standard duration of five weeks. Rehabilitation patients are admitted to the internal medicine department and undergo a comprehensive cardiological diagnosis. Psychosomatic admission is carried out by a medical or psychological reference therapist and is supplemented by various psychological tests. At the end of the diagnostic process, joint rehabilitation goals are developed with regard to physical and psychosocial health as well as participation in working life and society.

The program is comprised of mandatory elements, such as individual and group psychotherapy, exercise therapy, relaxation techniques and dietary consultation, as well as optional modules, such as creative therapies, physiotherapy and disease lectures. Hence, rehabilitants received a core program supplemented with facultative elements according to individual needs. Table 2 gives an overview of the modules of psychocardiological rehabilitation.

**Table 2:** Elements of psychocardiological rehabilitation.

General procedure	Admission by the cardiology ward physician and psychosomatic reference therapist One cardiology consultation and one interdisciplinary team meeting per week Team consultation with the chief physicians / senior physicians from both departments in the first week and after a further 2–3 weeks At least 2 × 30-min individual therapy sessions and 2 × 90-min indicative group sessions per week
Individual cognitive-behavioural therapy	Development of an individual disease model, taking into account the patient’s biography, learning history, cardiological findings, etc Presentation of correlations, using models (vicious circle model, etc.) if necessary Behavioral experiments and activation Work on cognitions
Medical consultation	Provision of cardiological information, discussion of diagnostic results, medication planning, etc
Complementary therapeutic elements	Psychoeducational seminars on cardiology and psychosomatics Sports and exercise therapy according to cardiological standards (4 × 30’ ergometer training/week with cardiovascular monitoring, gymnastics/water gymnastics depending on cardiac capacity, indicative strength/endurance training and (Nordic) walking Learning a relaxation technique (PMR) or qi gong Social counseling and social therapy Occupational therapy/cognitive training/art therapy (depending on indication) Integrated social medical assessment according to double specialist standard

An individual therapeutic exercise program was determined for each rehabilitation patient. The exercise therapy program consisted of a basic program, endurance training, and additional facultative interventions, e.g., respiratory therapy, yoga and Qi Gong. The basic program included body awareness training, muscular and balance training and mobilization (2 × 60 min/week). Endurance training was offered three times a week for 30 min in the form of controlled ergometer training and twice a week by walking. Training pulse was determined beforehand via exercise ECG. Progressive muscle relaxation according to Jacobson was performed in the short (30 min in a sitting position) and long form (45 min in a lying position). In addition, patients were trained in attention control, muscle tone reduction, influencing pulse and blood pressure and transfer of these new skills into everyday life. Psychotherapy included 2 × 30 min of individual therapy and 2 × 90 min of a cognitive-behavioral group therapy per week, focusing on interventions to manage anxiety and depression and to improve coping. Psychotherapy typically included psychoeducation (e.g. circle of fear, stress-vulnerability model), thought protocols, cognitive restructuring, behavioral activation, and behavioral experiments. Optional modules were art therapy and dance therapy. Furthermore, depending on their needs, rehabilitation patients received intensive social therapy support to clarify social, medical, and professional issues and develop return-to-work plans.

### Measures

Questionnaire measures were collected at admission (T0) and discharge (T1), sociomedical assessment was carried out ahead of discharge (T1).

#### Predictor variables

Heart anxiety was measured by the German version of the Cardiac Anxiety Questionnaire (CAQ), general anxiety and depressive symptoms were assessed by the Hospital Anxiety and Depression Scale (HADS-A and HADS-D, respectively). Delta scores were computed from scores at admission and discharge. Analyses included baseline scores from outcome measures.

#### Outcome variables

Subjective fitness to work was assessed by a questionnaire on activity and participation according to the International Classification of Functioning (MINI-ICF-APP). Objective fitness to work was determined by sociomedical assessment of work ability and participation impairments. Quality of life was evaluated by self-assessments of psychological and physical quality of life (Short Form—12, SF-12).

#### Depressive and general anxiety symptoms

*Hospital Anxiety and Depression Scale (HADS)*^30,31^. The HADS is a standardized self-rating instrument for assessing symptoms of general anxiety and depression in patients with physical complaints. The questionnaire has 14 items, which are rated on a four-point Likert scale, with a higher score indicating a higher symptom burden^32^. In the literature, values for Cronbach’s alpha for HADS-A varied from 0.68 to.93 (mean 0.83) and for HADS-D from 0.67 to 0.90 (mean 0.82)^33^. In the current investigation, Cronbach’s alpha was 0.78 for HADS-A and 0.89 for HADS-D. The HADS has demonstrated good construct and convergent validity, showing strong correlations with the Beck Depression Inventory (BDI) and the State-Trait Anxiety Inventory (STAI) (r = 0.60–0.80)^31,33^.

#### Heart anxiety symptoms

*Cardiac Anxiety Questionnaire (CAQ)*^34,35^. The CAQ is a standardized screening instrument for assessing heart-related anxiety in patients with cardiological diseases. 17 items are rated on a five-point Likert scale. A total score and three subscales (fear, avoidance, attention) are computed. Cronbach’s alpha for the CAQ is reported to vary between 0.69 and 0.92. In this study, Cronbach’s alpha was 0.86. High values indicate clinically relevant symptoms. The CAQ shows strong convergent validity with measures of general anxiety (HADS-A) and health anxiety (r = 0.55–0.70). Discriminant validity is demonstrated by weaker correlations with depression measures (r < 0.40), supporting its specificity for cardiac-focused anxiety. Criterion validity has been confirmed through significant associations with cardiac symptom perception, avoidance of physical exertion, and healthcare utilization.

#### Subjective and objective work ability

*MINI-ICF rating for activity and participation impairments in mental illness—self-rating (MINI ICF APP-S)*^36–38^. The Mini-ICF-APP-S is based on the International Classification of Functioning, Disability and Health (ICF) published by the World Health Organization. It is used to assess the extent to which a patient is impaired in the performance of activities and abilities. The following abilities are assessed: (1) Ability to adapt to rules and routines, (2) Ability to plan and structure tasks, (3) Flexibility and ability to adapt, (4) Skill and knowledge application, (5) Decision-making and judgement ability, (6) Proactivity and spontaneous activities, (7) Ability to resist and persevere, (8) Ability to assert oneself, (9) Ability to converse and socialize with others, (10) Ability to work in groups, (11) Ability to maintain close dyadic relationships, (12) Ability for self-care and self-sufficiency, (13) Mobility and transport skills. Higher values indicate greater impairments. The inter-rater-reliability of the MINI ICF ranged from 0.63 to 0.91. The MINI-ICF-APP-S has demonstrated good construct and convergent validity, showing expected correlations with measures of psychosocial functioning (e.g., Global Assessment of Functioning, GAF) and symptom severity (r = 0.50–0.75). Its criterion validity has been established by significant associations with clinicians’ judgments of work capacity and rehabilitation outcomes^39^.

The sociomedical assessment was performed by both senior specialists in cardiology and psychosomatic medicine honoring the dual control principle. For their rating of work capacity, information from all treatment units was compiled in team meetings. The procedure for the sociomedical assessment was based on the guidelines of the German Federal Pension Insurance^40^. At its core, the procedure begins with a comprehensive file review and clinical examination, followed by structured evaluation of disease-typical impairments in daily and professional life. The assessor must evaluate (a) the extent and nature of physical or psychological impairments, (b) the resulting restrictions on the claimant’s capacity for work in the labor market, and (c) whether rehabilitation measures are indicated to restore functional capacity. Throughout the procedure key standards of transparency, completeness and scientific validity apply, ensuring that the assessment adheres to the uniform criteria set out in the guidelines for sociomedical assessment and is traceable and reproducible in decision-making. The outcome of this process is an assessment of the patient’s ability to work in the general labor market, which may be full (> 6 h a day), reduced (< 6 h a day) or none (< 3 h a day). For this study, the latter two categories were combined to provide a binary outcome: full-time work capacity vs. reduced work capacity.

#### Quality of life

*Short Form Health Survey 12 (SF-12)*^41^. The SF-12 is a self-rating instrument for assessing the health-related quality of life. It is the short version of the SF-36^42^ and consists of 12 items, which are rated in different response formats. Physical and mental health-related quality of life scales can be determined individually. In the literature, intraclass correlation coefficients were ICC = 0.73 for the physical subscale and ICC = 0.80 for the mental subscale^43^. In our study, Cronbach’s alpha was 0.78 for the physical subscale and 0.83 for the mental subscale. Higher total scores reflect a better health-related quality of life. Regarding construct validity, higher physical and mental health scores were associated with higher odds of returning to work^44^.

### Statistical analyses

Statistical analyses were performed using R Version 4.3.2 and R Studio 2023.09.1 + 494. Demographic statistics were computed for age, sex, level of education (middle school or lower vs. high school or higher), sociomedical assessment (full-time vs. limited work ability) and psychological and cardiological diagnoses. Missing data were imputed using k nearest neighbors as implemented in the *VIM* package^45^ using all numeric EvaPK variables. Missing data rates are shown in S1.

Clinical and demographic characteristics were compared between patients with depressive disorders, anxiety disorders and comorbid depressive and anxiety disorders to determine potential covariates by ANOVA and Chi^2^ tests for continuous and categorical variables, respectively. In addition, S2 shows a Pearson correlation matrix for all binary and numeric variables.

Descriptive statistics were computed for all questionnaire measures at T0 and T1. Furthermore, paired sample t-Tests were run for each scale between T0 and T1 followed by analyses of Cohen’s D and confidence intervals to measure effect size.

Assessments of test assumptions can be found in the Supplementary Data. Normality of data was assessed by visual inspection of histograms (S3). Multicollinearity was tested for by looking at variance inflation factors (VIF). For multiple regressions, linearity was assessed by component and residual plots (S4), independence of errors by Durbin-Watson tests (S5), homoscedasticity by residuals vs fitted plots (S6), and normality of residuals by Q-Q plots and histograms of residuals (S7). For the logistic regression, influential observations were assessed by Cook’s distance (S8) and Goodness-of-fit by Hosmer–Lemeshow test (S9).

Improvements in symptom load were computed by subtracting T0 from T1 measurements of the HADS depression and anxiety subscales and the CAQ, respectively, to obtain delta scores. Z standardized delta scores (ΔHADS-D, ΔHADS-A, ΔCAQ) were then entered as predictors in individual multiple regressions with the MINI-ICF-APP-S total score and SF-12 physical and psychological quality of life subscale scores at T1 as response variables. Predictors of the binary sociomedical assessment outcome (full-time work capacity vs. reduced work capacity) were examined via logistic regression. All models included ICD-coded depression and anxiety, baseline symptom severity (z-standardized HADS-D, HADS-A, and CAQ scores) as well as the baseline score of the respective outcome where applicable (i.e. z-standardized scores of the MINI-ICF-APP-S, SF-12 mQoL and SF-12 pQoL). Model fit was evaluated using adjusted R^2^ and Tjur’s R^2^ for the logistic regression. For the logistic regression, coefficients were exponentiated to report odds ratios (ORs) with 95% confidence intervals (CIs) based on profile likelihood estimates. Results were considered statistically significant for *p*-values < 0.05.

## Results

Demographic characteristics of the sample are shown in Table 3. The sample had a mean age of 54.8 years (SD = 7.2) and was balanced regarding gender (51.0% female, 48.9% male). All participants reported at least one psychiatric diagnosis. Affective disorders were the most common (79.8%), followed by anxiety disorders (62.5%), adjustment disorders (8.6%), PTSD (9.5%), eating disorders (2.1%), and hypochondria (3.7%). Among cardiological diagnoses, 48.2% of patients had ischaemic heart disease, 32.5% conduction or arrhythmic disorders, 30.9% hypertension, and 26.8% structural defects, cardiomyopathy, or heart failure. Less frequent conditions included vascular diseases (11.1%), heart transplants or cardiac device implantation (9.1%), and other cardiac or circulatory diseases (6.2%). Most participants had completed middle school or a lower school form (70.8%), and three-quarters (75.3%) were assessed to have an earning capacity greater than 6 h per day.

**Table 3:** Demographic characteristics.

	N	M	SD	min	max
Age	243	54.83	7.15	31	69

		N	%
Sex	Female Male	124 119	51.0 48.9
Psychiatric diagnoses	Affective disorders Anxiety disorders Adjustment disorders PTSD Eating disorder Hypochondria	194 152 21 23 5 9	79.8 62.5 8.6 9.5 2.1 3.7
Cardiological diagnoses	Hypertension Ischaemic heart diseases Conduction /arrhythmic disorders Structural defects, cardiomyopathy, heart failure Vascular diseases Heart transplant or cardiac Device implant Other cardiac and circulatory diseases	75 117 79 65 27 22 15	30.9 48.2 32.5 26.8 11.1 9.1 6.2
Education level	Low (middle school or lower) High (high school or higher)	172 71	70.8 29.2
Earning capacity	< 6 h / day > 6 h / day	60 183	24.7 75.3

Abbreviations: N = sample size, M = mean value, SD = standard deviation, min = minimum value, max = maximum value.

Clinical and demographic characteristics by psychological diagnosis, i.e. for patients with depressive disorders (n = 91), anxiety disorders (n = 49), and comorbid depressive and anxiety disorders (n = 103) are shown in Table 4. The groups did not differ significantly regarding age (F = 1.99, *p* = 0.14), sex (χ^2^ = 2.68, *p* = 0.26) or education level (χ^2^ = 4.9, *p* = 0.09). Earning capacity differed significantly between groups (χ^2^ = 11.8, *p* = 0.003), with patients with anxiety disorders more often assessed as being able to work full-time compared to the other groups. In addition, patients with anxiety disorders showed the lowest scores on all predictor measures except the CAQ at both admission and discharge, while patients with comorbid disorders showed the highest symptom levels on HADS-D, HADS-A and CAQ. All group differences for these clinical measures were statistically significant (F-values = 13.45–35.17, *p* ≤ 0.001).

**Table 4:** Demographic and clinical characteristics by psychological diagnosis.

Measures	Depressive disorders (n = 91)	Anxiety disorders (n = 49)	Comorbid depressive and anxiety disorders (n = 103)		
	M (SD)			F	*P*
HADS-D T0	10.4 (4.75)	5.27 (2.91)	11.1 (4.11)	35.17	** ≤ 0.001**
HADS-A T0	9.95 (3.90)	8.31 (3.11)	11.7 (3.35)	16.28	** ≤ 0.001**
CAQ T0	1.54 (0.60)	1.80 (0.52)	2.08 (0.58)	21.11	** ≤ 0.001**
HADS-D T1	6.87 (4.50)	3.69 (2.94)	8.47 (4.58)	20.75	** ≤ 0.001**
HADS-A T1	7.04 (3.72)	5.73 (3.41)	9.54 (4.03)	19.91	** ≤ 0.001**
CAQ T1	1.35 (0.57)	1.38 (0.51)	1.75 (0.62)	13.45	** ≤ 0.001**
Age	55.5 (6.7)	53.1 (7.6)	55 (7.3)	1.99	0.14

	N			χ^2^	*P*
Sex Female Male	41 50	29 20	54 49	2.68	0.26
Education level Low High	72 19	32 17	68 35	4.9	0.09
Earning capacity < 6 h / day > 6 h / day	24 67	3 46	32 70	11.8	**0.003**

Abbreviations: n = size of subsample, F = F-statistic, χ^2^ = Chi^2^ statistic, *P* = *p*-value, M = mean score, SD = standard deviation, HADS-D T0 = Hospital Anxiety and Depression Scale—Depression at admission, HADS-A T0 = Hospital Anxiety and Depression Scale—Anxiety at admission, CAQ T0 = Cardiac Anxiety Questionnaire at admission, HADS-D T1 = Hospital Anxiety and Depression Scale – Depression at discharge, HADS-A T1 = Hospital Anxiety and Depression Scale—Anxiety at discharge, CAQ T1 = Cardiac Anxiety Questionnaire at discharge. Statistically significant results are indicated in bold.

Descriptive statistics, paired t-Tests and effect sizes of questionnaire responses at T0 and T1 are depicted in Table 5. Higher values indicate greater impairment on the HADS, CAQ and MINI-ICF-APP-S questionnaires and greater well-being on the SF-12 scales. Across all measures, paired-samples t-tests indicated significant changes from T0 to T1 (all *p* ≤ 0.001). Depressive (HADS-D) and anxiety symptoms (HADS-A) both showed large reductions from T0 to T1 (Cohen’s d = −0.73 and −0.71, respectively). Participants also reported lower cardiac anxiety (CAQ; d = −0.59) and slightly improved functional capacity (MINI-ICF; d = −0.34). Physical and mental quality of life improved significantly, with medium-to-large effect sizes (d = 0.52 and d = 0.61, respectively). Confidence intervals confirmed the robustness of the observed effects as none included zero.

**Table 5:** Descriptive statistics.

Measures	T0				T1				Mean Diff	paired t-Test			Cohen’s D	
	M	SD	Min	Max	M	SD	Min	Max	T1 – T0	T	Df	*P*	D	CI
HADS-D	9.66	4.71	0	20	6.91	4.6	0	21	− 2.76	− 11.35	242	**≤ 0.001**	− 0.73	[− 0.87, − 0.59]
HADS-A	10.35	3.74	1	18	7.84	4.08	0	19	− 2.51	− 11.35	242	**≤ 0.001**	− 0.71	[− 0.85, − 0.57]
CAQ	1.82	0.62	0.29	3.29	1.52	0.61	0.12	3.24	− 0.3	− 11 − 11	242	**≤ 0.001**	− 0.59	[− 0.73, − 0.45]
MINI-ICF-APP-S	2.56	0.97	0.31	6.15	2.31	0.92	0	5.77	− 0.25	− 5.37	242	**≤ 0.001**	− 0.34	[− 0.47, − 0.21]
SF-12 pQoL	38.43	9.54	14.03	63.44	42.3	9.88	19.39	60.04	3.87	8.06	242	**≤ 0.001**	0.52	[0.38, 0.65]
SF-12 mQoL	35.43	9.87	15.76	59.32	41.1	11.19	11.34	64.82	5.67	9.47	242	**≤ 0.001**	0.61	[0.47, 0.74]

Abbreviations: T0 = admission, T1 = discharge, M = mean score, SD = standard deviation, min = minimum score, max = maxima score, T = T statistic, df = degrees of freedom, *P* = *p*-value, D = Cohen’s D, CI = Confidence Interval for Cohen’s D, HADS-D = Hospital Anxiety and Depression Scale—Depression subscale, HADS-A = Hospital Anxiety and Depression Scale—anxiety subscale, CAQ = Cardiac Anxiety Questionnaire, MINI-ICF-APP = MINI-ICF Assessment of activity and participation impairments in mental illness—self-rating, SF-12 pQOL = Short Form-12 physical Quality of Life, SF-12 mQoL = Short Form-12 mental Quality of Life. Statistically significant results are indicated in bold.

Multiple regression analyses are shown in Table 6. We found that self-rated work impairment as assessed by the MINI-ICF-APP-S at T1 was significantly predicted by improvements in general anxiety symptoms as assessed by ∆ HADS-A (β = 0.14, *p* = 0.03) and improvements in heart anxiety symptoms as assessed by ∆ CAQ (β = 0.11, *p* = 0.03). Significant covariates were baseline measures MINI-ICF-APP-S T0 (β = 0.46, *p* ≤ 0.001), HADS-D T0 (β = 0.20, *p* ≤ 0.001) and CAQ T0 (β = 0.10, *p* = 0.05). The overall model accounted for 62% of the variance in work impairment (adj. R^2^ = 0.62). Variance inflation factors ranged between 1.34 (MINI-ICF T0) and 2.91 (∆ HADS-A).

**Table 6:** Multiple regression analyses.

Measures	Beta	SE	df	T	*P*
MINI-ICF-APP-S T1					
ICD Depression ICD Anxiety MINI-ICF T0 HADS-D T0 HADS-A T0 CAQ T0 ∆ HADS-D ∆ HADS-A ∆ CAQ	0.19 0.03 0.46 0.20 0.09 0.10 0.09 0.14 0.11	0.12 0.09 0.04 0.06 0.06 0.05 0.06 0.06 0.05	233 233 233 233 233 233 233 233 233	1.65 0.33 10.87 3.63 1.92 2.02 1.49 2.23 2.32	0.09 0.74 ** ≤ 0.001** ** ≤ 0.001** 0.09 **0.05** 0.11 **0.03** **0.03**
adj. R^2^ = .62					
SF-12 pQoL T1					
ICD Depression ICD Anxiety SF-12 pQoL T0 HADS-D T0 HADS-A T0 CAQ T0 ∆ HADS-D ∆ HADS-A ∆ CAQ	− 0.50 1.28 0.63 − 0.62 − 0.19 − 1.16 − 1.18 − 1.18 − 2.12	1.26 0.97 0.05 0.59 0.61 0.56 0.62 0.68 0.55	233 233 233 233 233 233 233 233 233	− 0.40 1.32 13.59 − 1.05 − 0.31 − 2.08 − 1.89 − 1.73 − 3.87	0.69 0.19 ** ≤ 0.001** 0.29 0.76 **0.04** 0.06 0.09 ** ≤ 0.001**
adj. R^2^ = .61					
SF-12 mQoL T1					
ICD Depression ICD Anxiety SF-12 mQoL T0 HADS-D T0 HADS-A T0 CAQ T0 ∆ HADS-D ∆ HADS-A ∆ CAQ	− 1.70 0.42 0.43 − 2.11 − 2.19 0.03 − 1.15 − 3.76 − 1.59	1.44 1.09 0.07 0.71 0.73 0.62 0.69 0.76 0.61	233 233 233 233 233 233 233 233 233	− 1.19 0.39 5.99 − 2.96 − 2.99 0.05 − 1.64 − 4.96 − 2.6	0.24 0.69 ** ≤ 0.001** **0.003** **0.003** 0.96 0.10 ** ≤ 0.001** **0.009**
adj. R^2^ = .62					

Abbreviations: ICD Depression = Diagnosis of a depressive disorder (ICD-10), ICD Anxiety = Diagnosis of an anxiety disorder (ICD-10, HADS-D T0 = Hospital Anxiety and Depression Scale—Depression at admission, HADS-A T0 = Hospital Anxiety and Depression Scale—Anxiety at admission, CAQ T0 = Cardiac Anxiety Questionnaire at admission, ∆ HADS-D = Mean difference in HADS-D between T0 and T1, ∆ HADS-A = Mean difference in HADS-A between T0 and T1, ∆ CAQ = Mean difference in CAQ between T0 and T1, Beta = standardized regression coefficient, SE = Standard error, df = degrees of freedom, T = T statistic, *P* = *p*-value. Statistically significant results are indicated in bold.

Physical QoL (SF-12) at T1 was significantly predicted by ∆ CAQ (β = −2.12, *p* ≤ 0.001) and by covariates SF-12 pQoL T0 (β = 0.63, *p* ≤ 0.001) and CAQ T0 (β = −1.16, *p* = 0.04). The model explained 61% of the variance in physical quality of life at T1 (adjusted R^2^ = 0.61). Variance inflation factors ranged between 1.25 (SF-12 pQoL T0) and 2.95 (∆ HADS-A).

Mental QoL (SF-12) at T1 was significantly predicted by ∆ HADS-A (β = −3.76, *p* ≤ 0.001) and ∆ CAQ (β = −1.59, *p* ≤ 0.01). Significant covariates were SF-12 mQoL T0 (β = 0.43, *p* ≤ 0.001), HADS-D at T0 (β = −2.11, *p* = 0.004) and HADS-A at T0 (β = −2.19, *p* = 0.003). The model accounted for 62% of the variance in psychological quality of life (adjusted R^2^ = 0.62). Variance inflation factors ranged between 1.42 (ICD anxiety) and 2.90 (∆ HADS-A).

The logistic regression analysis is given in Table 7. Higher baseline depressive symptoms (HADS-D T0) were significantly associated with increased odds of reduced work capacity as opposed to full-time work capacity (OR = 1.78, 95% CI [1.15, 2.80], *p* = 0.01). None of the other predictors, including ICD-coded depression, ICD-coded anxiety, HADS-A, CAQ, or change scores, reached statistical significance (all *p* > 0.05). Adjusted Tjur’s R^2^ for the logistic regression was 0.11. Variance inflation factors ranged between 1.24 (ICD Depression) and 2.56 (∆ HADS-A).

**Table 7:** Logistic regression analysis.

Measures	Beta	SE	df	Z	*P*	OR	95% CI (OR)
Reduced work capacity							
ICD Depression ICD Anxiety **HADS-D T0** HADS-A T0 CAQ T0 ∆ HADS-D ∆ HADS-A ∆ CAQ	1.29 0.12 0.57 − 0.10 0.09 0.15 0.27 0.27	0.69 0.38 0.23 0.23 0.22 0.24 0.28 0.23	241 241 241 241 241 241 241 241	1.85 0.33 2.54 − 0.43 0.40 0.63 0.95 1.22	0.06 0.74 **0.01** 0.67 0.69 0.53 0.34 0.22	3.62 1.13 1.78 0.90 1.09 1.16 1.31 1.32	[1.03, 17.18] [0.54, 2.38] [1.15, 2.80] [0.57, 1.43] [0.72, 1.67] [0.72, 1.89] [0.76, 2.29] [0.85, 2.06]
adj. R^2^ = 0.11							

Abbreviations: ICD Depression = Diagnosis of a depressive disorder (ICD-10), ICD Anxiety = Diagnosis of an anxiety disorder (ICD-10), HADS-D T0 = Hospital Anxiety and Depression Scale—Depression at admission, HADS-A T0 = Hospital Anxiety and Depression Scale—Anxiety at admission, CAQ T0 = Cardiac Anxiety Questionnaire at admission, ∆ HADS-D = Mean difference in HADS-D between T0 and T1, ∆ HADS-A = Mean difference in HADS-A between T0 and T1, ∆ CAQ = Mean difference in CAQ between T0 and T1, Beta = standardized regression coefficient, SE = Standard error, df = degrees of freedom, Z = Z statistic, *P* = *p*-value, OR = Odds ratio, 95% CI (OR) = 95% confidence interval for odds ratios. Statistically significant results are indicated in bold.

## Discussion

The present study investigated the association of symptom improvements in anxiety and depression on sociomedical outcome, subjective work impairments and quality of life in patients with comorbid cardiological diseases and depressive or anxiety disorders following psychocardiological rehabilitation.

We found symptom load to be highest in patients who meet criteria for both anxiety and depressive disorders and lowest in patients with anxiety but not depression. This was also reflected in sociomedical assessments at discharge from rehabilitation, which saw patients with anxiety most likely to work full-time. Notably, symptom improvements as such were similarly high for anxious and depressive symptoms, and slightly lower for cardiac anxiety symptoms. In addition, patients diagnosed with depressive disorders showed highest improvements in both anxious and depressive symptoms, whereas patients with anxiety disorders improved most regarding cardiac anxiety symptoms. Also, baseline depression scores were significantly associated with higher subjective work impairments and lower mental quality of life as well as a higher likelihood of being assessed as having reduced work capacity at discharge. We can therefore conclude that depressive disorders both play a major role in the capacity to pursue full-time employment and respond well to rehabilitative interventions in heart-disease patients. Yet, these improvements were not statistically predictive of the functional rehabilitation outcomes investigated in this study. Instead, reductions in general and heart-focused anxiety significantly predicted lower self-assessed work impairments and better mental quality of life at discharge, and improvements in cardiac anxiety symptoms were associated with better physical quality of life.

These results require careful and differentiated considerations. First off, they are in line with previous studies reporting that depression plays a pivotal role in limiting participation in daily activities and work^46^, and more so than anxiety^47,48^, as baseline depression scores were significantly associated with both subjective and objective limitations in work capacity. Moreover, depression has previously been reported as risk factor for poorer functional recovery, reduced adherence, and worse prognosis in cardiac populations^10,49^.

Our results further confirm previous findings reporting a lack in translation of improved depressive symptoms to improved cardiac and functional outcomes. Improvements in overall depressive symptoms were found to fail in improving cardiac event-free survival in coronary heart disease patients, with the authors suggesting residual symptoms of anhedonia, fatigue and insomnia as hindering factors^50^. A “paradox of symptom remission and functional recovery” was also observed in non-cardiological populations with major depression, meaning that symptom improvements did not predict functional recovery^51^. Improvements in anxious symptoms on the other hand have been found to directly relate to improved health behaviors, such as exercise or health-care utilization, and improved quality of life^52–54^. Heart patients with elevated levels of anxiety often exhibit avoidance behaviors, heightened physiological responses, and impaired concentration, which can directly affect their ability to return to work or engage in physical activity^55,56^. That is, anxiety may exert more immediate and physiologically reactive effects on daily functioning in cardiac patients, which is why symptom improvement turns more easily into functional gains.

More persistent biological disruptions characteristic of depressive disorders may further help explain why improvements in depressive symptoms don’t necessarily translate into functional recovery. Major depression has been associated with structural and neuroplastic alterations such as hippocampal atrophy, reduced synaptic density, and glial dysfunction that can endure even after symptoms abate^57–59^, whereas anxiety disorders typically involve more reversible hyperreactivity of threat-processing circuits^60,61^. Depression is also related to chronic dysregulation of the hypothalamic–pituitary–adrenal axis and low-grade inflammation that persist into remission and may adversely affect cognition and motivation, as well as occupational functioning^62–65^. Further, deficits in reward processing and motivation due to mesolimbic dopamine system dysfunction may contribute to reduced engagement and effort in daily activities even after mood improves^66,67^. These enduring biological disturbances provide a plausible explanation for why functional recovery in depression often lags behind symptom remission, whereas anxiety disorders show more rapid normalization of underlying neurobiological systems.

### Clinical implications

The significant association between reductions in anxiety symptoms and functional outcomes indicates that anxiety-focused interventions (e.g. cognitive-behavioral therapy, relaxation training) may have direct and measurable benefits for daily functioning and occupational reintegration. Meanwhile, the results underscore that improvements in depressive symptoms may not directly translate into functional recovery, despite comparable responsivity to treatment. This dissociation between symptom remission and functional recovery may warrant further adjustments in psychocardiological rehabilitation programs. Complementary strategies targeting anhedonia, motivational impairments, residual cognitive deficits and biological dysregulation may be required to support full functional recovery. These might include Acceptance and Commitment (ACT) therapy, mindfulness-based interventions, or pharmacological strategies aimed at enhancing neuroplasticity and reward processing.

### Limitations

While the results reported in our investigation are corroborated by previous findings, several caveats should be addressed regarding their interpretation. First, they are based on the data of a single clinic and rely on pre-post observational measures, which limits interpretability in terms of generalizability and causation. In addition, the sample was predominantly middle-aged, with a significant proportion of participants having a low education level, which may further limit the generalizability of the findings to other age groups or more diverse populations. Moreover, the rehabilitation program encompassed optional modules, which may have been used disproportionately. It would be interesting to look at the specific elements of psychocardiological rehabilitation in order to identify potentially impactful interventions. In addition, the reliance on self-report measures, such as the HADS and SF-12, introduces the potential for bias, particularly given the subjective nature of symptom assessments in patients with comorbid psychological and physical conditions. Future studies could benefit from including objective measures of physical capacity and functional performance to complement self-reported outcomes. The explanatory power of the logistic regression was comparatively low at R^2^ = 0.11, which however can be expected for an outcome of this complexity level. Finally, we did not conduct an analysis of sample size needed to detect significant effects prior to the analyses, although this had been done for the EvaPK study protocol.

## Conclusions

Our results showed that while depressive and anxiety symptoms are similarly responsive to psychocardiological rehabilitation, only the latter are related to improved functional outcomes. Future research should therefore explore the impact of additional modules targeting refractory depressive symptoms, which may close the gap between symptom improvement and functional restitution. Overall, our study also highlights the need for consideration of functional factors and quality of life as outcome measures of health interventions^68,69^.

## Supplementary Information

Below is the link to the electronic supplementary material.

Supplementary Material 1

## Data Availability

The datasets generated and analyzed during the current study are available from the corresponding author on request.
