# Supplementary material for: Improvement of anxious but not depressive symptoms is associated with functional outcomes of psychocardiological rehabilitation
Source: Sci Rep. 2025 Dec 26;15:44640. doi: 10.1038/s41598-025-32594-7 (PMC12749061; doi:10.1038/s41598-025-32594-7)
Supplement: Supplementary file 1 — Supplementary Material 1 [file 41598_2025_32594_MOESM1_ESM.pdf]

Supplementary Data

S1. Missingness analysis

S1. Missing data

variable	N missing
sociomedical assessment	1
MINI-ICF-APP-S T0	2
MINI-ICF-APP-S T1	33
SF-12 mQoL T0	0
SF-12 mQoL T1	15
SF-12 pQoL T0	0
SF-12 pQoL T1	15
HADS-A T0	4
HADS-A T1	26
HADS-D T0	4
HADS-D T1	26
CAQ T0	0
CAQ T1	14

Abbreviations: N missing = number of missing data entries, T0 = admission, T1 = discharge, HADS-D = Hospital Anxiety and Depression Scale – Depression subscale, HADS-A = Hospital Anxiety and Depression Scale – anxiety subscale, CAQ = Cardiac Anxiety Questionnaire, MINI-ICF-APP = MINI-ICF Assessment of activity and participation impairments in mental illness - self-rating, SF-12 pQOL = Short Form-12 physical Quality of Life, SF-12 mQoL = Short Form-12 mental Quality of Life

S2. Pearson correlation matrix of numeric and binary study variables

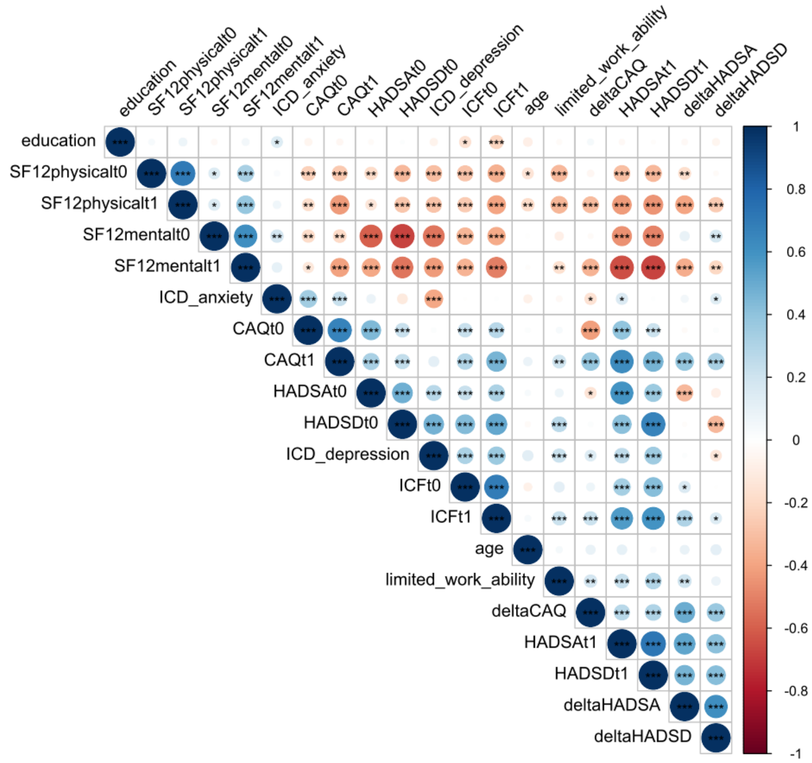

S3. Assessment of Normality (Histograms)

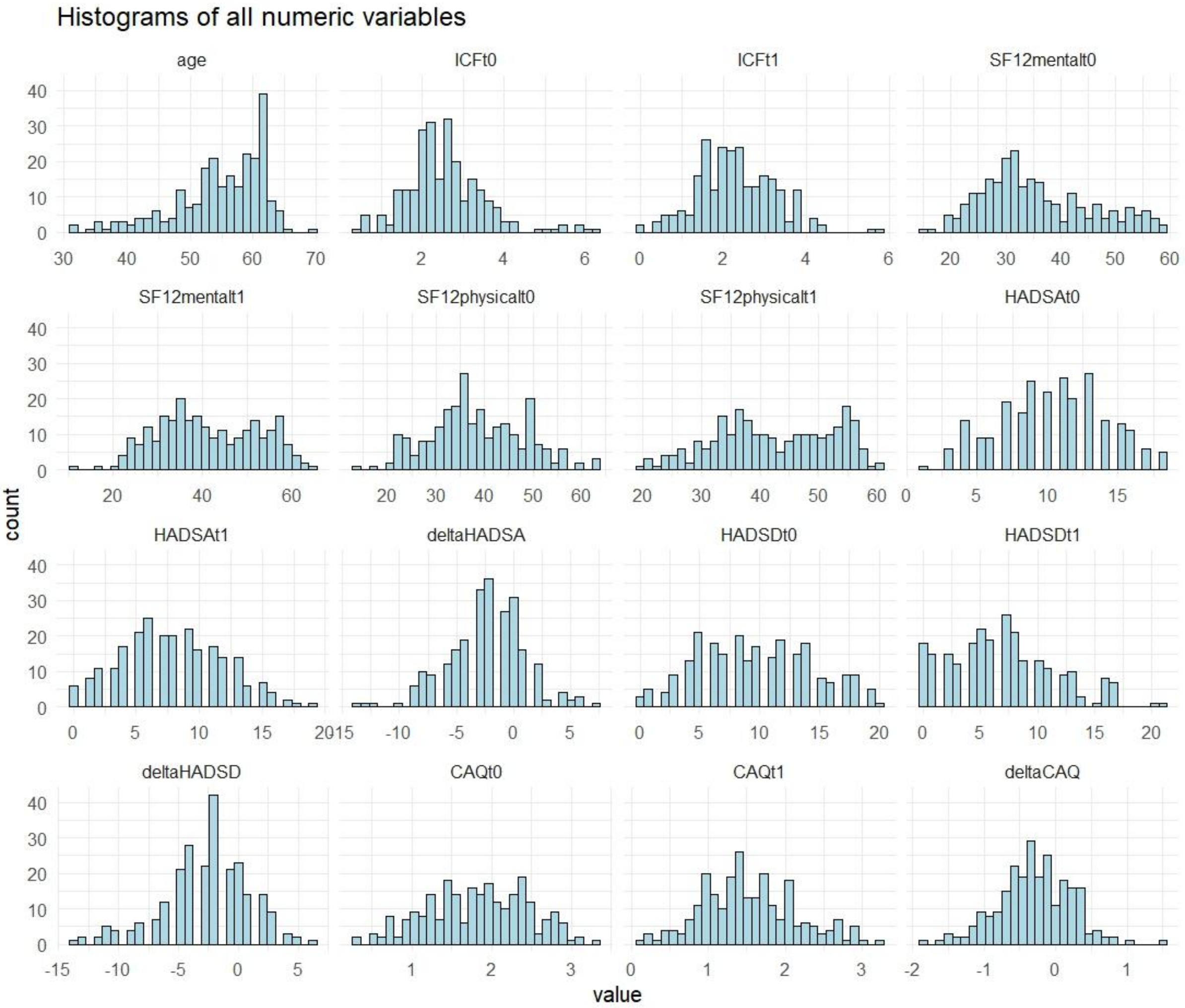

S4. Assessment of Linearity (Component and Residual plots)

MINI-ICF-APP-S

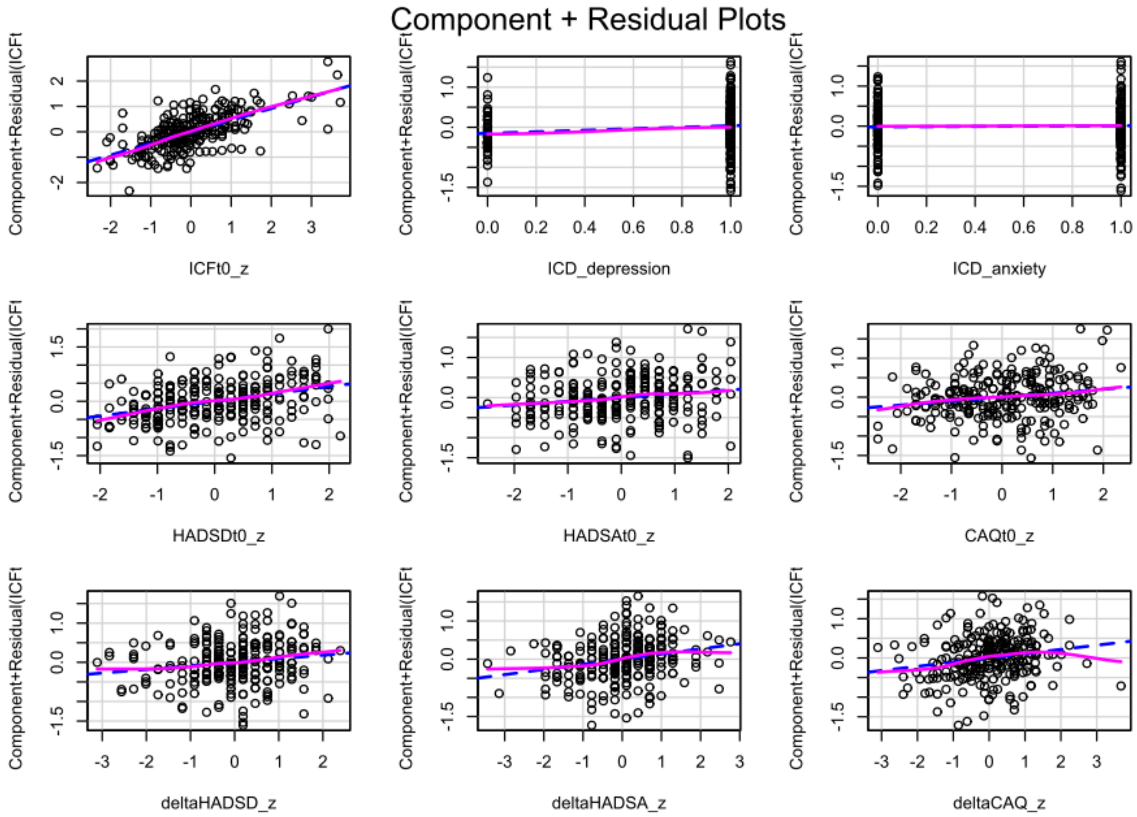

SF-12 pQoL

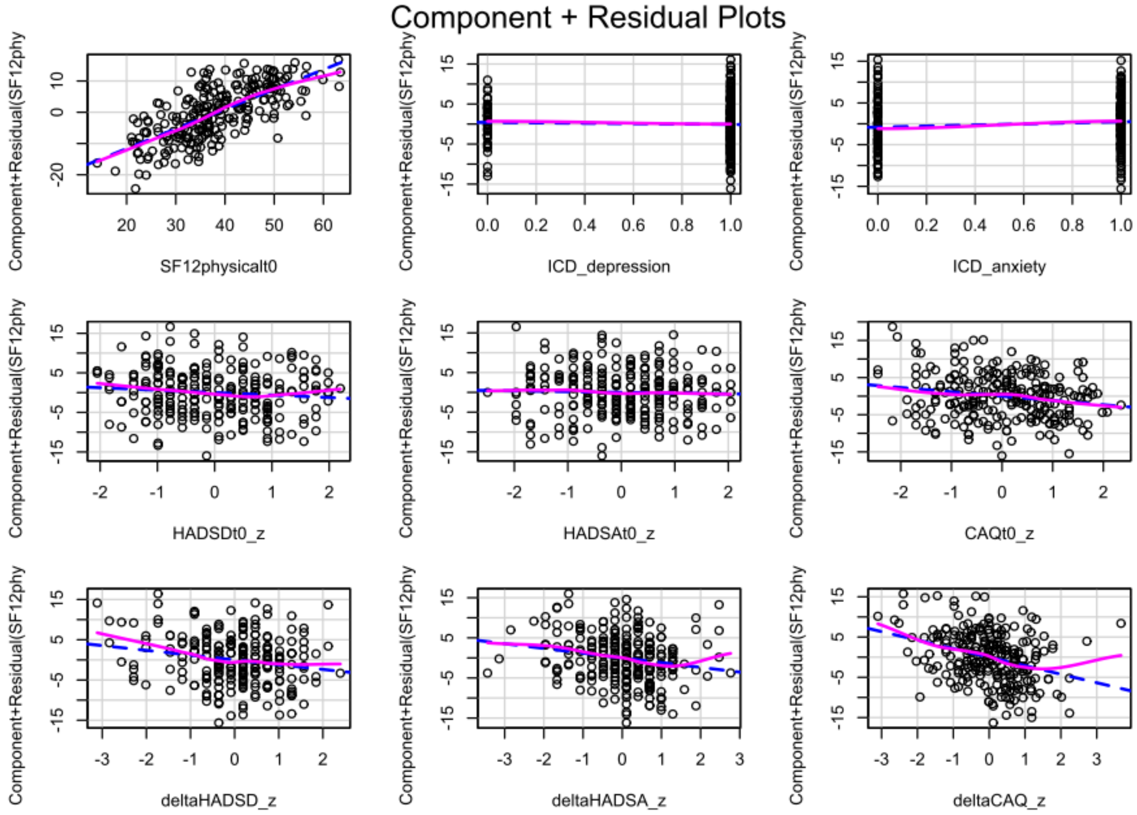

SF-12 mQoL

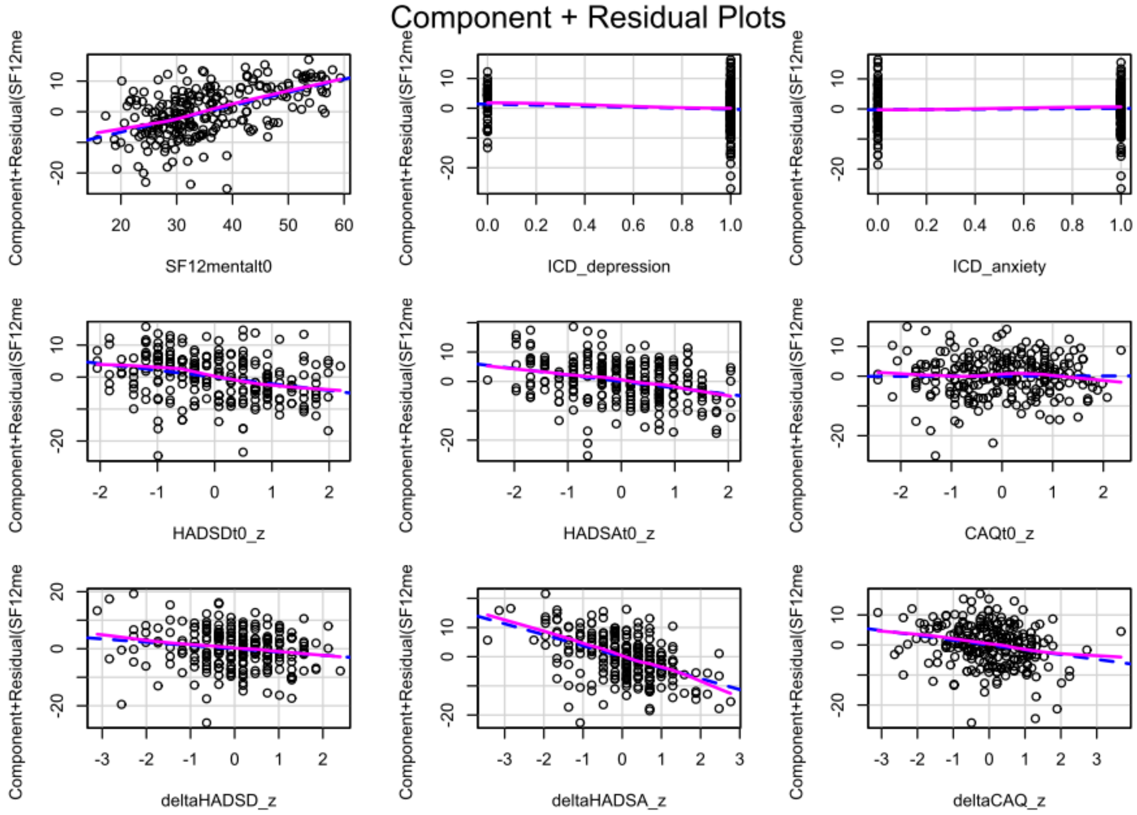

Component + Residual Plots
Component+Residual(SF12me
SF12mentalt0
ICD_depression
ICD_anxiety
HADSDt0_z
HADSAt0_z
CAQt0_z
deltaHADSD_z
deltaHADSA_z
deltaCAQ_z

S5. Assessment of independence of residuals (Durbin-Watson tests)

The independence of residuals in the regression models was evaluated using the Durbin–Watson test. For all outcomes (MINI-ICF-APP-S, SF-12 pQoL, and SF-12 mQoL), the Durbin–Watson statistics were close to 2 (MINI-ICF-APP-S: 1.85, $p = 0.24$; SF-12 pQoL: 1.95, $p = 0.67$; SF-12 mQoL: 2.24, $p = 0.052$), indicating no significant first-order autocorrelation. These results suggest that the assumption of independent residuals was met for all models.

S6. Assessment of Homoscedasticity (Residuals vs Fitted plots)

MINI-ICF-APP-S

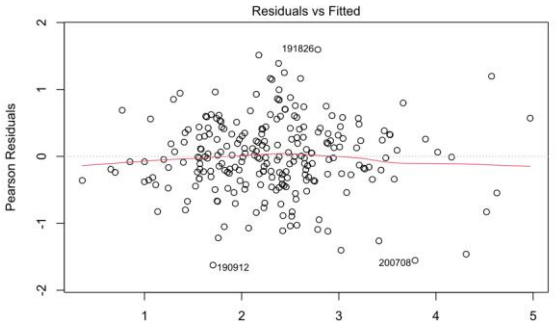

SF-12 pQoL

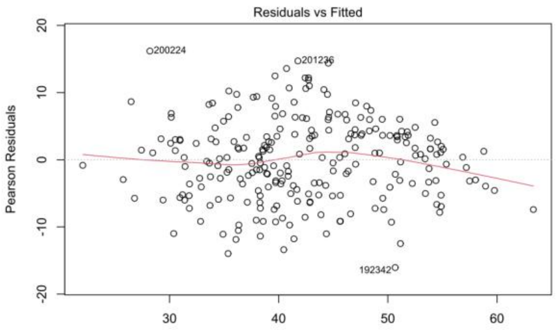

SF-12 mQoL

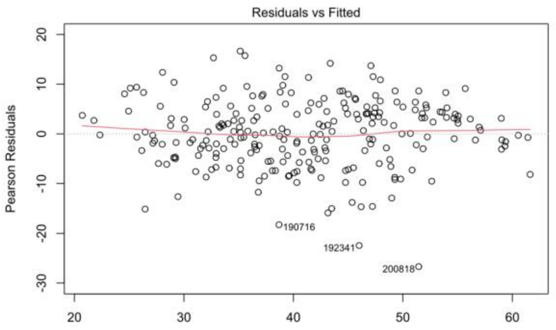

S7. Assessment of Normality of Residuals (Q-Q plots and Histograms of Residuals)

MINI-ICF-APP-S

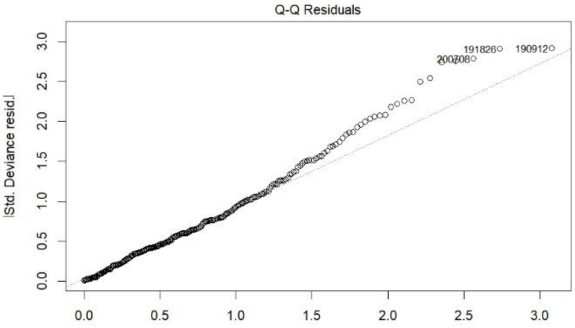

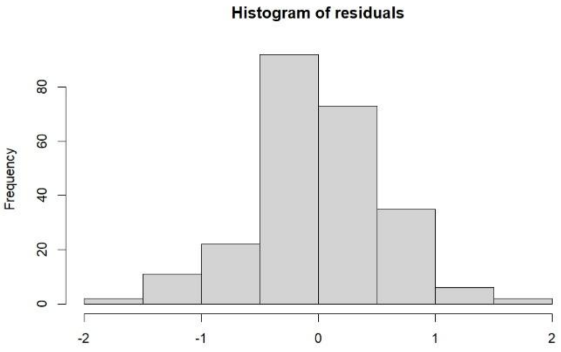

SF-12 pQoL

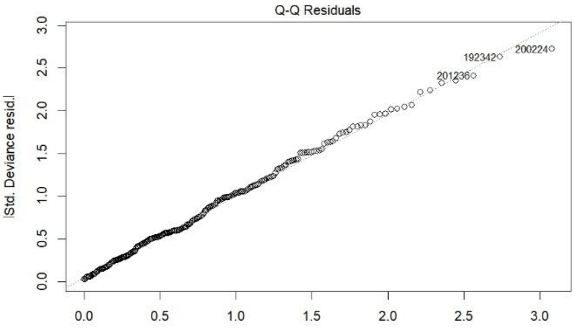

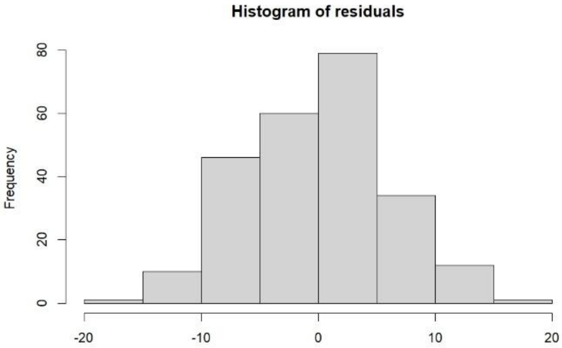

SF-12 mQoL

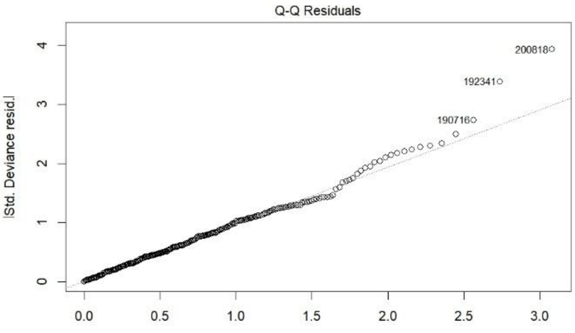

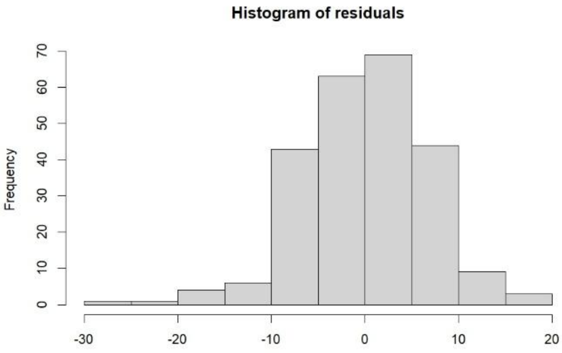

S8. Assessment of influential observations (Cook's distance and Hosmer-Lemeshow test)

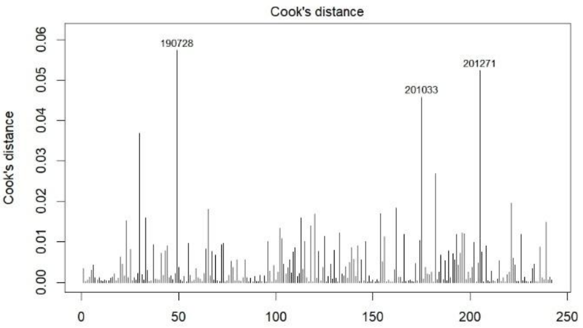

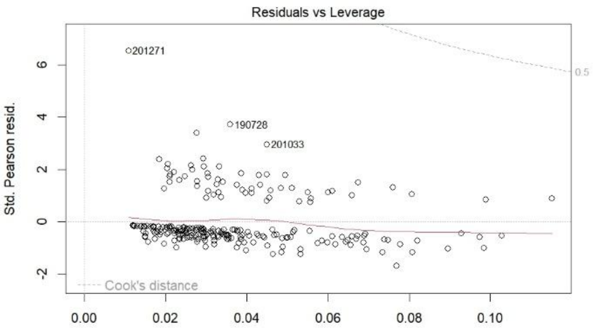

S9. Assessment of Goodness-of-fit (Hosmer-Lemeshow test)

The goodness-of-fit of the logistic regression model was assessed using the Hosmer–Lemeshow test. The results indicated that the model fit the data well ($\chi^2 = 4.98$, df = 8, $p = 0.76$), suggesting no significant deviation between the observed and predicted outcomes.
